# Oxidative stress decreases the redox ratio and folate content in the gut microbe, *Enterococcus durans* (MTCC 3031)

**DOI:** 10.1038/s41598-018-30691-4

**Published:** 2018-08-14

**Authors:** Steffi Jose, Prerna Bhalla, G. K. Suraishkumar

**Affiliations:** Department of Biotechnology, Bhupat and Jyoti Mehta School of Biosciences building Indian Institute of Technology Madras, Chennai, 600036 India

## Abstract

Gut microbiome plays an important role in determining the effectiveness of cancer therapy. The composition of the microbiome is crucial to maintain good digestive health in the host, and to prevent and treat colorectal cancers. Most cancer therapies employ oxidative stress, which disturbs the redox status of the cell, and consequently affect growth, reductive biosynthesis and cell death. Therefore, oxidative stress can undesirably affect the gut microbiome. Hence, it is important to understand the impact of oxidative stress on gut bacteria to devise effective treatment strategies. The current study induces oxidative stress in the model gut bacterium *Enterococcus durans* (MTCC 3031) with menadione and H_2_O_2_. Oxidative stress considerably decreased the redox ratio (NADPH/NADP), an indicator of the redox status, by 55% (menadione) and 28% (H_2_O_2_). In addition, an oxidative stress induced decrease in redox ratio decreased folate synthesis by the bacteria, which is an undesirable consequence for the host, since folate deficiency can induce colorectal cancer. Further, oxidative stress considerably decreased growth and the biomass density by 61% (menadione) and 21% (H_2_O_2_). Thus, maintenance of the cellular redox status and management of oxidative stress in the gut microbiome may be crucial to the effectiveness of cancer treatment strategies.

## Introduction

It was recently established that the gut microbiota significantly impact the efficacy of cancer treatment in humans^1^. It is possible that they play a role in determining the body’s response to cancer immunotherapy^1^. Also, the gut microbiota synthesize and supply the host with folate, the deficiency of which induces colorectal cancers^2^. Further, they are involved in the regulation of the host’s antioxidant response through the modulation of reduced glutathione (GSH) synthesis^3^.

On the other hand, the gut microbiota can themselves be subjected to oxidative stress^4^ during cancer treatment with redox drugs. Most chemotherapeutic drugs kill cancer cells by inducing high oxidative stress^5,6^ and such drugs, when they reach the gut, can cause oxidative stress in the gut microbiota. The oxidative stress, in turn, can alter the intracellular redox status of the gut microbiota. Since the intracellular redox status is associated with several important functions ranging from growth, reductive biosynthesis of various metabolites to apoptosis^7^, disturbances in the redox status could lead to an altered microbiota, which are unable to perform their regular functions for the host. The redox status can be represented with the NADPH/NADP ratio or the redox ratio^8^. Therefore, it is important to understand the effect of oxidative stress on intracellular redox ratio in the gut microbiota. Such an understanding could contribute to improved management of associated diseases. Also, it could lead to better effectiveness of new cancer therapies based on redox signaling^6^, and redox signalling, in turn, is effected by reactive species^9^. Redox signalling based cancer therapies are expected to overcome multi-drug resistance associated with normal chemotherapy^10^.

The objective of this study is to demonstrate in principle, the impact of high oxidative stress on a ‘model’ gut bacterium, a situation that can arise in cancer therapy due to the induction of oxidative stress in these therapies^5,6,11^. The non-pathogenic gut bacterium *Enterococcus durans* (MTCC 3031) was used as the model organism for this study. It is one among the several bacteria that comprise the gut microbiota^12,13^. We found that oxidative stress decreased the cellular redox status in *E*. *durans* (MTCC 3031). We have also shown that there is a redox-ratio related decrease in the production of the anti-cancer molecule, folate, by the model gut bacterium; the result has significance in effective chemotherapy planning, especially for colorectal and stomach cancers. In addition, oxidative stress also significantly decreased bacterial growth, an effect that could negatively alter the gut microbiota.

## Results and Discussion

### Inherent oscillatory nature of redox ratio during batch growth

**Figure 1:**
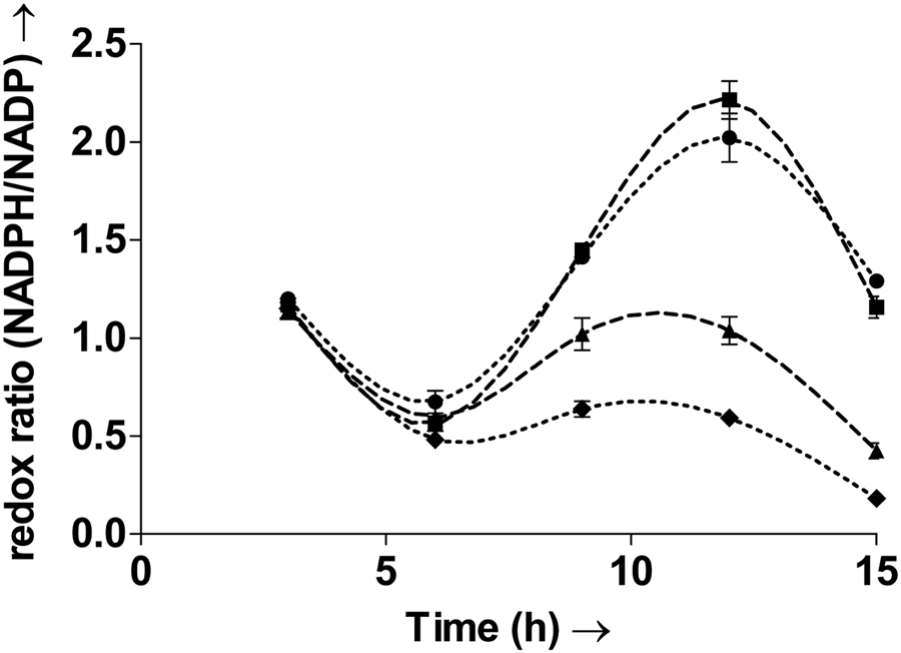
Temporal variation in the whole cell redox ratio (NADPH/NADP) of the control culture C (∙∙●∙∙), culture H treated with H_2_O_2_ (**–**▲–), culture H + A treated with H_2_O_2_ followed by the antioxidant mannitol (–■–) and culture M treated with menadione (∙∙∙♦∙∙∙). The redox ratio of the cell oscillates with time. Exposure to oxidative stress leads to decrease in the redox ratio. The ratio clearly decreases after the introduction of oxidative stress in cultures H, H + A and M. Culture C refers to control. Oxidative stress was induced in the bacterial cultures H and M with pulses of H_2_O_2_ and menadione, respectively, after 5.5 h and 11.5 h. The culture H + A was treated with H_2_O_2_ after 5.5 h and then with the antioxidant mannitol after 8.5 h. Values are expressed as mean ± SD, n = 3.

It is known that oxidative stress can perturb redox homeostasis^14^. To characterize the impact of oxidative stress on the redox status of the gut microbe *E. durans* (MTCC 3031), the whole cell redox ratio (NADPH/NADP) was measured. Temporal measurements of the redox ratio, of all the cultures are plotted in Fig. 1. The whole cell redox ratio (NADPH/NADP) is said to be maintained a constant in studies on eukaryotic cells^8^. However, measurements in the bacterium here clearly show that the ratio oscillates with time. Oscillations in the ratio have not been previously observed in bacteria. The results show that the redox ratio varies widely with time. The values range from 0.67 ± 0.05 to 2.02 ± 0.1 even within the control culture. This indicates that the oscillatory nature is an inherent property of the bacterial cell.

### Oxidative stress considerably decreases the redox ratio

Oxidative stress induced with pulses of menadione and H_2_O_2_ led to substantial decreases in the redox ratio, as seen from Fig. 1. Since the ratio is important for several functions including reductive biosynthesis, its decrease would undesirably impact these functions.

The oscillatory nature and trends of the redox ratios in H (H_2_O_2_) and M (menadione) were however, similar to that of control. Following the pulse addition of menadione and H_2_O_2_, after 5.5 hours, the redox ratio measured after the 6^th^ hour showed a 29% and 12% decrease in M and H respectively, from the control ratio of 0.67 ± 0.05. The redox ratios decreased even further after 9 hours. The ratio was lower than control by 55% and 28% in M and H respectively. A second pulse addition of menadione and H_2_O_2_ was carried out after 11.5 hours. This additional induction of oxidative stress led to even further decreases in the redox ratio. After the 12^th^ hour, the redox ratio was lower than control by 70% and 49% in M and H, respectively. Whereas after the 15^th^ hour, the ratio was lower than control by 86% and 67% in M and H, respectively. It was observed that, although the bacterial cultures were exposed to the same concentrations of H_2_O_2_ and menadione, the latter induced a more severe decrease in the redox ratio. Increases or decreases in the ratio previously observed via single point measurements in bacteria have been suggested to have significant impact on metabolic decisions^15,16^. Therefore, such significant decreases in the ratio due to oxidative stress could adversely affect cell metabolism. Further, rhythms observed in NADPH levels of eukaryotic photosynthetic systems have been suggested to have links with the circadian rhythms^17^. Thus, the oscillatory nature observed in the ratio may also play a role in maintaining the biological clock in bacteria.

### The hydroxyl radical affects the redox ratio

**Figure 2:**
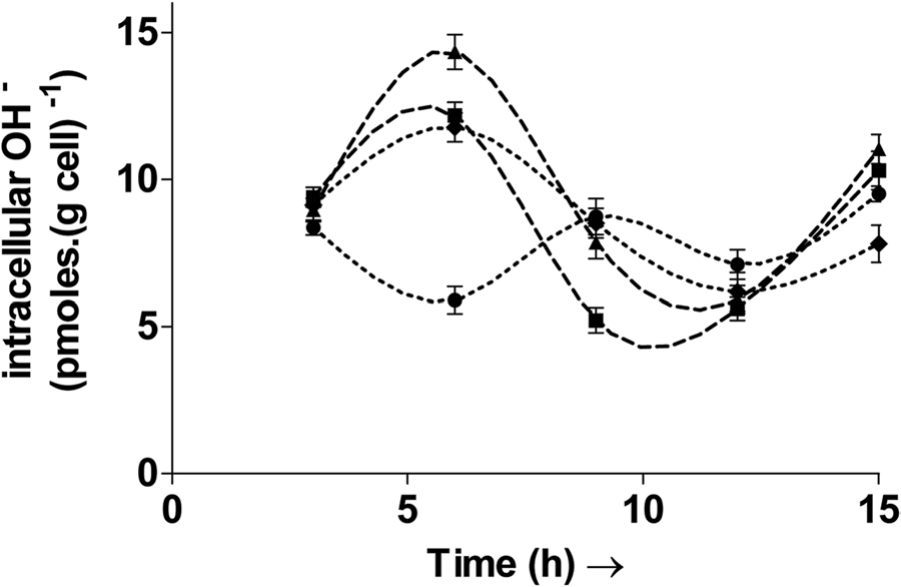
Temporal variation of intracellular OH radical levels in the control culture C (∙∙●∙∙), culture H treated with H_2_O_2_ (–▲–), culture H + A treated with H_2_O_2_ followed by the antioxidant mannitol (–■–) and culture M treated with menadione (∙∙∙♦∙∙∙). The intracellular hydroxyl radical levels oscillate with the time. Inducing oxidative stress in cultures H, H + A and M led to an initial increase in hydroxyl radical levels over control (C). Oxidative stress was induced in the bacterial cultures H and M with pulses of H_2_O_2_ and menadione, respectively, after 5.5 h and 11.5 h. The culture H + A was treated with H_2_O_2_ after 5.5 h and then with the hydroxyl radical scavenger mannitol after 8.5 h. Values are expressed as mean ± SD, n = 3.

The hydroxyl radicals are highly reactive oxygen species (hROS) and a common manifestation of oxidative stress^18^. It can cause extensive damage in biomolecules due to its high instability^19^. It reacts rapidly (within nanoseconds of formation) and non-specifically with numerous organic molecules thereby causing oxidative damage^18^. Thus, the intracellular concentration of this ROS was measured in all cultures and is shown in Fig. 2. The OH^−^ concentration was found to vary with time and exhibited an oscillatory nature. Fluctuations and rhythms in “total ROS’ have been previously identified but limited to eukaryotes such as fungi^20^, plants^21^, insects^22^ etc., and are involved in the transcriptional regulation of select genes. The oscillations observed here indicate that hydroxyl radicals may also have rhythms in the bacterial cell. The introduction of H_2_O_2_ and menadione pulses after 5.5 hours in M, H and H + A (H_2_O_2_ followed by mannitol) led to relative increases in the OH^−^ concentration, as observed after the sixth hour. Such an increase is possible since H_2_O_2,_ following uptake by the cell can lead to the generation of hydroxyl radicals via the Fenton reaction^23^. Menadione also leads to the generation of free radicals, during its metabolism by the cell^16^.

To see if the hydroxyl radical formation specifically affected the redox ratio, the antioxidant mannitol - an OH– scavenger, was added to the culture H + A. The redox ratio in H + A after six hours was similar to H due to the addition of H_2_O_2._ However, the ratio recovered to that of control after 9 hours following the addition of mannitol in H + A. The culture H + A recorded a redox ratio of 1.44 ± 0.03 while that in H remained at just 1. Similarly the redox ratio in H + A after 12 hours increased to 2.21 ± 0.09, comparable to control, whereas that in H was still at a lower value of 1.03 ± 0.06. Thus, the hydroxyl radicals can directly affect the redox ratio and addition of mannitol was able to reverse the adverse effects of H_2_O_2_ (at the concentration used in the study), on the ratio.

### Decreased folate levels during oxidative stress: Increased host susceptibility to colorectal cancer

Folate synthesis by the gut bacterium is one of the many major functions it provides for the host since the human body cannot synthesize folate on its own. Inadequate supplementation or deficiency of folate has been associated with the development of colorectal cancers in humans^2^. Therefore, the impact of oxidative stress on intracellular folate levels was studied.

**Figure 3:**
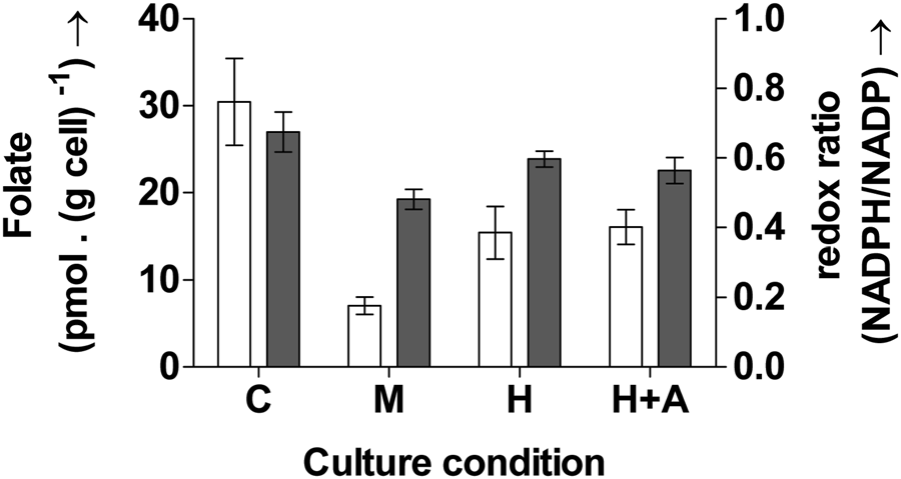
Intracellular folate levels (open bars) and the corresponding redox ratio (closed bars) measured after 6 h in the control culture C, culture H treated with H_2_O_2_, culture H + A treated with H_2_O_2_ followed by the antioxidant mannitol and culture M treated with menadione. Oxidative stress induction led to significant decreases in both folate and the redox ratio. Culture C refers to control. Oxidative stress was induced in the bacterial cultures with pulses of H_2_O_2_ (H and H + A) and menadione (M), after 5.5 h. Values are expressed as mean ± SD, n = 3. One way ANOVA of the intracellular folate levels and the redox ratio showed that differences in the values of the different cultures were significant with *p* < 0.001 for intracellular folate and *p* = 0.002 for redox ratio. Tukey’s multiple comparisons test showed that redox ratio levels in H + A and M and folate levels in all the cultures were significantly different from control.

Exposure to oxidative stress in the current study led to substantial decreases in folate content as shown in Fig. 3. This would consequently leave the host more prone to cancer. Folate measurement for the current study was carried out only once: after six hours. This time point was chosen because previous studies in the lab showed that folate accumulation in the bacterium was highest between 6–7 hours in the organism. Further, a single measurement requires a high cell concentration (10 OD) for accurate and reproducible measurements.

The control culture showed the highest folate concentration of 30.4 ± 5 pmoles (g cell)^−1^. Exposure to both H_2_O_2_ and menadione led to significant decreases in folate content (*p* < 0.001, one-way ANOVA with α = 0.05). The folate content was only 7 ± 1 pmoles (g cell)^−1^ in M; 77% lower than control whereas the addition of H_2_O_2_ led to a decreased folate content of 15.4 ± 3 pmoles (g cell)^−1^.

In addition to the folate content, the corresponding redox ratios of the different cultures, measured after the sixth hour is also shown in Fig. 3. The figure shows simultaneous increase/decrease of the folate content with the redox ratio. A decrease in the redox ratio in H and M is accompanied by similar decreases in the folate content in the respective cultures.

Folate is essential for DNA synthesis^24,25^. Decreased folate content can thus obstruct growth of the organism. This importance is in fact targeted in cancer therapy^26^. It is also used in the development of antibiotics that disrupt folate metabolism and consequently arrest bacterial growth and infection^25^. Exposure to oxidative stress in the current study led to decreases in both folate content and growth in this study. The reduced folate content may have also contributed to the decreased growth and biomass productivity observed here (detailed in the following section).

### Decreased growth: Oxidative stress can alter the microbiome

**Figure 4:**
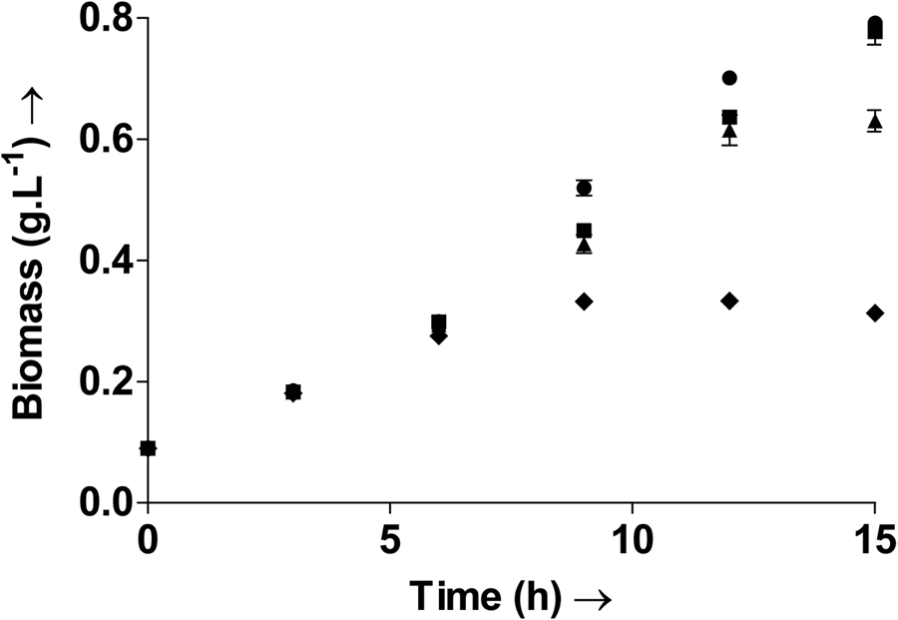
Time profile of the biomass density in the control culture C (●), culture H treated with H_2_O_2_ (▲), culture H + A treated with H_2_O_2_ followed by the antioxidant mannitol (■) and culture M treated with menadione (♦). Oxidative stress led to decreased growth and biomass density. Oxidative stress was induced in the bacterial cultures H and M with pulses of H_2_O_2_ and menadione, respectively, after 5.5 h and 11.5 h. The culture H + A was treated with H_2_O_2_ after 5.5 h and then with the hydroxyl radical scavenger mannitol after 8.5 h. Values are expressed as mean ± SD, n = 3.

The impact of oxidative stress on the growth and biomass production of the gut microbe was studied and severe decreases were observed. Biomass accumulated in the cultures was measured at regular intervals and are shown in Fig. 4. The highest biomass accumulation was observed with the control (C) cultures, as expected. Oxidative stress induced by menadione (M) and H_2_O_2_ (H and H + A) led to substantial decreases in the biomass accumulation. No visible lag phase was recorded. The exponential phase extended right upto the 15^th^ hour in C and H + A. The cultures H and M, however, entered the stationary phase in the 12^th^ hour. The maximum specific growth rate, measured during the logarithmic phase of each culture was comparable in the cultures C, H and H + A with a value of 0.15 h^−1^, whereas that in M was lower at 0.13 h^−1^. Following the pulse addition after 5.5 hours, the biomass measured at the 6^th^ hour was comparable in all cultures. However, further growth measurements displayed the adverse effects of the oxidative agents on the culture growth. The biomass density measured after 9 hours was only 0.33 g L^−1^ and 0.42 g L^−1^ in M and H respectively, compared to the control density of 0.5 g L^−1^. Although the biomass density in H and H + A were comparable at this time point, addition of the antioxidant mannitol to H + A led to increases in growth as can be seen in the subsequent measurements. As a result the biomass density of H + A is comparable to C at the 15^th^ hour, whereas the cultures M and H show a 61% and 21% decrease respectively. As already discussed earlier, decrease in folate under oxidative stress may have also additionally contributed to the decreased growth and biomass production in these cultures.

Growth rate may also be directly related to the redox ratio. Maximum biomass accumulation rates coincided with rapid increases in the redox ratio. This is explained with Supplementary Fig. S1. Although the exponential growth phase extends up to the 15^th^ hour in C, the biomass accumulation ‘rates’, the total biomass formed per unit time, measured for each time interval varies. Supplementary Fig. S1 indicates the period/time interval during which maximum biomass production was actually obtained for a particular culture. The maximum biomass accumulation ‘rate’ in C, was recorded between the 6^th^ and 9^th^ hours (0.07 g L^−1^ h^−1^), and then the 9^th^ and 12^th^ hours (0.06 g L^−1^ h^−1^) as shown in Supplementary Fig. S1. The highest increases in the biomass accumulation rate in cultures H and H + A were recorded between hours 9 and 12, followed by that between 6 and 9 hours. It can be seen here that the redox ratio shows a drastic increase between the 6^th^ and 12th hours - the same period during which biomass accumulation rates were highest (Supplementary Fig. S1). Thus a decrease in the redox ratio could directly impact growth.

A decrease in the population of the gut microbe would indicate an altered microbiota. Balance in the gut flora is already known to be essential for good health and a disturbance in its composition and population has been associated with inflammatory bowel diseases^4,27^. Disturbance of the gut microbiota due to the administration of antibiotics is already known to cause several undesirable consequences in patients^28^. Similarly, oxidative stress, if not contained, can lead to several disturbances, including the development of diseases in the host. Oxidative stress mediated damage to the gut microbiota has already been linked to severe acute malnutrition (SAM) in humans^29^. Therefore, therapies employing oxidative stress will have to be devised so as to minimize oxidative stress in gut microbiome to improve treatment efficacy and avoid other harmful consequences.

In conclusion, results of the current study show that oxidative stress in the gut bacteria *Enterococcus durans* (MTCC 3031) can lead to severe decreases in the cellular redox ratio, intracellular folate content and cell growth. Such an effect would be detrimental to the host. The results have relevance in treatments that expose the gut bacteria to oxidative stress, such as in cancer therapies. Decreased redox ratio decreases the reductive power of the cell and would affect a range of cellular functions. Decreased folate synthesis by the bacteria can lead to folate deficiency in the host, a condition that can induce colorectal cancer. Decreased bacterial growth would lead to a significant reduction in the bacterial population under such circumstances. It is already known that maintenance of the cellular redox status is crucial for proper functioning of the gut microbiome. This study further indicates that the efficacy of treatment strategies that expose the bacteria to oxidative stress, such as in cancers and infections, could be greatly reduced due to altered gut microbiota. A balance towards this effect could be the key to more effective treatment strategies. Maintenance of the cellular redox status is thus imperative for devising effective treatment strategies.

## Methods

### Organism and culture

The gut bacterium *Enterococcus durans* (MTCC 3031), was purchased from MTCC. An overnight culture of *E. durans* (MTCC 3031), in MRS media was used as the inoculum. The organism was cultured in a Scigenics Orbitek shaker incubator at 37 °C, 180 rpm.

### Inducing oxidative stress

Culture was carried out in 250 ml MRS broth. All flasks were inoculated to 0.2 OD for the study. The bacterium was cultured in four different conditions and labelled C, H, M and H + A. The organism cultured in MRS broth was treated as control (C). The bacterium was exposed to two different sources of oxidative stress, by pulse addition of the oxidative agents H_2_O_2_ and menadione, in equal concentrations, to separate cultures labelled H and M respectively. Pulse additions were carried out after 5.5 h and 11.5 h. The agents were added such that the final concentration of H_2_O_2_ and menadione (due to the pulse addition alone) in the media was 300 µM (after 5.5 h) and 5 mM (after 11.5 hours). Another culture, labelled H + A, maintained to study the effect of the antioxidant mannitol, was exposed to 300 µM after 5.5 hours and then with mannitol (1 mM final concentration) after 8.5 hours. The culture period was limited to 15 hours for the current study since previous batch experiments in the lab showed that the growth entered stationary phase after this period. Measurements of the various analytes were carried out at intervals of three hours.

#### Growth

Growth was followed with optical density (OD) measurements of the culture at 600 nm. One ml of the culture was pelleted at 8000 *g* and the pellet washed in saline. The washed pellet was further re-suspended in saline and its OD measured at 600 nm.

#### Hydroxyl radicals

Measurement of the radical was carried out using the dye Aminophenyl fluorescein (APF; Invitrogen^TM^ Molecular Probes^®^, CA) based on previously established protocols^30^. Cell concentration was normalised to 1 OD and used for the measurement to obtain ‘specific’ intracellular values. Cell pellet corresponding to 1 OD ml^−1^ was washed and re-suspended in saline. APF was added to the suspension to a final concentration of 10 µM, incubated at room temperature for 30 minutes and the fluorescent measurements carried out on a 96 well plate reader (LS 55, PerkinElmer, Liantrisant, UK) with excitation at 490 nm and emission at 515 nm.

#### Extraction of NADP(H) and redox ratio measurement

Redox ratio here is defined as the ratio of the amount of total NADPH to total NADP. The reduced (NADPH) and oxidized (NADP) forms of the nucleotide were extracted separately and measured according to Queval and Noctor, 2007^31^. Culture volume corresponding to 5 OD was used for extraction and measurement of each of the pyridine nucleotides. Cell pellet obtained by centrifuging the appropriate volume (corresponding to 5 OD) was washed with distilled water and re-suspended in 0.5 ml of 0.2 N HCl (for NADP) or 0.5 ml of 0.2 N NaOH (for NADPH) and lysed using a probe sonicator (Q700, Qsonica, LLC, Connecticut, USA), operated at 70% amplitude for a total process time of 4 min (pulse on and off time being 2 s). The lysate was centrifuged at 8000 *g* for 5 min and the supernatant incubated in boiling water for one minute. The extract was then rapidly cooled on ice and pH adjusted to 5–6 (for NADP) or pH 7–8 (for NADPH) for measurement via enzyme assay in a 96-well microplate. The reaction mixture in each well contained 100 µl sample, 60 µl of 0.1 M HEPES, pH 7.5, 2 mM EDTA; 20 µl of 1.2 mM DCPIP (dichlorophenolindophenol); 10 µl of 10 mM glucose-6-phospate; 10 µl of 20 mM PMS (phenazine methosulfate). The reaction was started by adding 10 µl of glucose-6-phosphate dehydrogenase (100 U.ml^−1^) and the decrease in absorbance at 600 nm was measured over 10 minutes. The amount of the reduced and oxidised pyridine nucleotides were deduced from standard plots constructed with pure NADP and NADPH using the same assay.

#### Folate extraction and measurement

Folate extraction and measurement protocols were adapted from previously established methods^32^. Bacterial culture corresponding to 10 OD was used for folate estimation. The pellet obtained on centrifuging the required volume was suspended in 1 ml DI water. The cells were lysed using a probe sonicator, as detailed for redox measurement. The lysed sample was then placed in a water bath at 100 °C for 5 minutes to release any folate bound to the Folate Binding Proteins (FBPs). This lysate was then centrifuged and the supernatant used for folate estimation via HPLC.

Separation and identification of folate was done using HPLC (Prominence, Shimadzu, Japan). The analytical column used was C–18, 5–µm LC column, 250 × 4.6 mm (00G–4252–E0, Phenomenex, Hyderabad, India). The mobile phase, 15% HPLC grade methanol in 0.05 M KH_2_PO_4_, was filtered (0.22 micron) and degassed prior to use. A linear solvent programme was used. Mobile phase flow rate was maintained at 0.4 mL.min^−1^. Folate was identified using a PDA detector (SPD–M20A, Shimadzu, Japan) at 295 nm. Samples were also filtered (0.22 micron) before loading into the column. The concentration of folate in the sample was deduced from a standard plot constructed using varying concentrations of pure folic acid.

### Statistical analysis

All cultures and measurements were carried out in triplicates (three biological replicates, each subjected to three technical replicates). Values have been reported as mean ± SD. One way ANOVA (level of significance = 0.05) and Tukey’s multiple comparisons test was carried out using Minitab 16.

## Electronic supplementary material

Supplementary Information

## Data Availability

All data generated or analysed during this study are included in this article (and its Supplementary Information files).

## References

[1] Gopalakrishnan V (2018). Gut microbiome modulates response to anti–PD-1 immunotherapy in melanoma patients. Science..

[2] Kim Y (2003). Role of Folate in Colon Cancer Development and Progression. J. Nutr..

[3] Mardinoglu A (2015). The gut microbiota modulates host amino acid and glutathione metabolism in mice. Mol. Syst. Biol..

[4] Morgan XC (2012). Dysfunction of the intestinal microbiome in inflammatory bowel disease and treatment. Genome Biol..

[5] Halliwell, B. & Gutteridge, J. M. C. Reactive species and disease: fact, fiction or filibuster? In *Free Radicals in Biology and Medicine* 488–613 (OUP Oxford, 2007, 2007).

[6] Wondrak GT (2009). Redox-Directed Cancer Therapeutics: Molecular Mechanisms and Opportunities. Antioxid. Redox Signal..

[7] Ivarsson R (2005). Redox control of exocytosis: Regulatory role of NADPH, thioredoxin, and glutaredoxin. Diabetes.

[8] Zhang J (2015). Determination of the Cytosolic NADPH/NADP Ratio in Saccharomyces cerevisiae using Shikimate Dehydrogenase as Sensor Reaction. Sci. Rep..

[9] D’Autréaux B, Toledano MB (2007). ROS as signalling molecules: Mechanisms that generate specificity in ROS homeostasis. Nat. Rev. Mol. Cell Biol..

[10] Polimeni M, Gazzano E (2014). Is redox signaling a feasible target for overcoming multidrug resistance in cancer chemotherapy?. Front. Pharmacol..

[11] Holmström KM, Finkel T (2014). Cellular mechanisms and physiological consequences of redox-dependent signalling. Nat. Rev. Mol. Cell Biol..

[12] Collins MD, Jones D, Farrow JAE, Kilpper-Balz R, Schleifer KH (1984). Enterococcus avium nom. rev., comb. nov.; E. casselipavus norn. rev., comb. nov.; E. durans norn. rev., comb. nov.; E. gallinarum comb. nov.; and E. malodoratus. Int. J. Syst. Bacteriol..

[13] Hudson, C. R. & Hiott, L. M. Anomalies in species identification of enterococci from veterinary sources using a commercial biochemical identification system. 245–250 (2003). 10.1046/j.1472-765x.2003.01302.x 12641720

[14] Trachootham D, Lu W, Ogasawara MA, Nilsa R-DV, Huang P (2008). Redox regulation of cell survival. Antioxid. Redox Signal..

[15] Steele Reynolds T (2017). ROS Mediated Selection for Increased NADPH Availability in Escherichia coli. Biotechnol. Bioeng..

[16] Singh R, Mailloux RJ, Puiseux-Dao S, Appanna VD (2007). Oxidative stress evokes a metabolic adaptation that favors increased NADPH synthesis and decreased NADH production in Pseudomonas fluorescens. J. Bacteriol..

[17] Zhou M (2015). Redox rhythm reinforces the circadian clock to gate immune response. Nature.

[18] Cohn CA, Pedigo CE, Hylton SN, Simon SR, Schoonen MAA (2009). Evaluating the use of 3′-(p-Aminophenyl) fluorescein for determining the formation of highly reactive oxygen species in particle suspensions. Geochem. Trans..

[19] Halliwell B, Chirico S (1993). Lipid peroxidation: its mechanism and significance. Am. J. Clin. Nutr..

[20] Yoshida Y, Iigusa H, Wang N, Hasunuma K (2011). Cross-talk between the cellular redox state and the circadian system in neurospora. PLoS One.

[21] Lai AG (2012). CIRCADIAN CLOCK-ASSOCIATED 1 regulates ROS homeostasis and oxidative stress responses. Proc. Natl. Acad. Sci..

[22] Krishnan N, Davis AJ, Giebultowicz JM (2008). Circadian regulation of response to oxidative stress in Drosophila melanogaster. Biochem. Biophys. Res. Commun..

[23] Bienert GP, Schjoerring JK, Jahn TP (2006). Membrane transport of hydrogen peroxide. Biochim. Biophys. Acta.

[24] Rossi M, Amaretti A, Raimondi S (2011). Folate production by probiotic bacteria. Nutrients.

[25] Murima P, McKinney JD, Pethe K (2014). Targeting bacterial central metabolism for drug development. Chem. Biol..

[26] Hagner N, Joerger M (2010). Cancer chemotherapy: Targeting folic acid synthesis. Cancer Manag. Res..

[27] Hullar MAJ, Burnett-Hartman AN, Lampe JW (2014). Gut Microbes, Diet, and Cancer. Cancer Treat. Res..

[28] de Gunzburg, J. *et al*. Protection of the human gut microbiome from antibiotics. *J. Infect. Dis*. **217** (2017). 10.1093/infdis/jix604 PMC5853327 29186529

[29] Million M (2016). Increased Gut Redox and Depletion of Anaerobic and Methanogenic Prokaryotes in Severe Acute Malnutrition. Sci. Rep..

[30] Setsukinai K, Urano Y, Kakinuma K, Majima HJ, Nagano T (2003). Development of novel fluorescence probes that can reliably detect reactive oxygen species and distinguish specific species. J. Biol. Chem..

[31] Queval G, Noctor G (2007). A plate reader method for the measurement of NAD, NADP, glutathione, and ascorbate in tissue extracts: Application to redox profiling during Arabidopsis rosette development. Anal. Biochem..

[32] Lin M, Young C (2000). Folate levels in cultures of lactic acid bacteria. Int. Dairy J..

