# Supplementary material for: Oxidative stress decreases the redox ratio and folate content in the gut microbe, Enterococcus durans (MTCC 3031)
Source: Sci Rep. 2018 Aug 14;8:12138. doi: 10.1038/s41598-018-30691-4 (PMC6092354; doi:10.1038/s41598-018-30691-4)
Supplement: Supplementary file 1 — Supplementary Information [file 41598_2018_30691_MOESM1_ESM.pdf]

Supplementary Information

Oxidative stress decreases the redox ratio and folate content in the gut microbe, *Enterococcus durans*

Steffi Jose, Prerna Bhalla and G. K. Suraishkumar*
Department of Biotechnology, Bhupat and Jyoti Mehta School of Biosciences building
Indian Institute of Technology Madras, Chennai 600036

***Correspondence to:**
Prof. G.K.Suraishkumar
Department of Biotechnology, Bhupat and Jyoti Mehta School of Biosciences building
Indian Institute of Technology Madras, Chennai 600036 India
E-mail: gk@iitm.ac.in
Phone: +914422574105
Fax: +914422574102

Supplementary figures

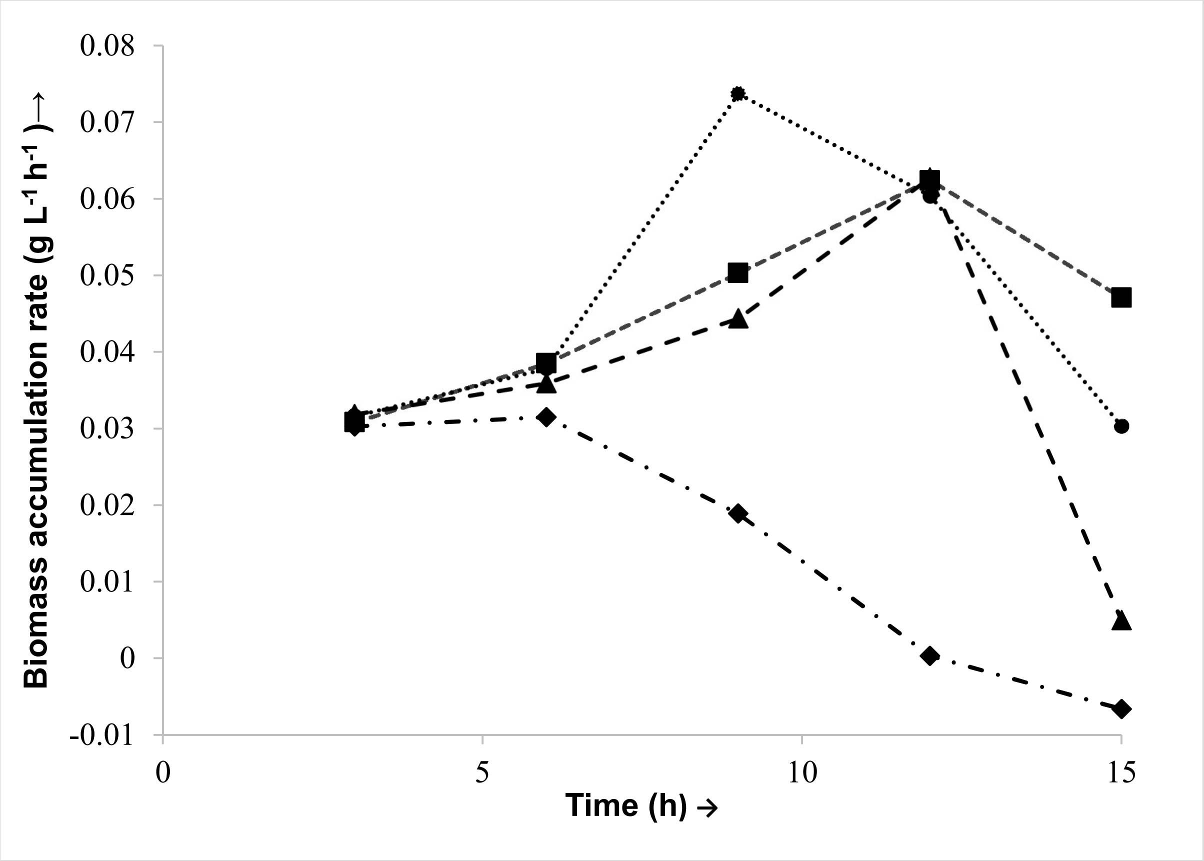

Supplementary Fig. S1: Biomass accumulation rate for each time interval of the control culture C (··●··), culture H treated with H_2O_2 (—▲—), culture H+A treated with H_2O_2 followed by the antioxidant mannitol (— ■ —) and culture M treated with menadione (· –♦– ·). The figure helps identify the period during which maximum biomass production was achieved for the various cultures. 'Change' in biomass density is displayed here. Positive values denote increase in biomass density whereas negative values arise from a decrease in the biomass density.
